# Tensor fascia lata free flap as salvage solution minimizes donor site morbidity in anterolateral thigh free flap failure—decision making algorithm and a case report

**DOI:** 10.3389/fsurg.2025.1692511

**Published:** 2025-11-13

**Authors:** Christian Soemmer, Ákos Bicsák, Evangelos N. Vitkos, Phillipp Brockmeyer, Stefan Haßfeld, Lars Bonitz

**Affiliations:** 1 Department of Oral- and Maxillofacial Surgery, Regional Plastic Surgery, Dortmund General Hospital, Dortmund, Germany; 2 Faculty of Health, University Witten/Herdecke, Witten, Germany; 3 Department of Oral- and Maxillofacial Surgery, University Medical Center Goettingen, Goettingen, Germany

**Keywords:** anterolateral thigh flap, salvage free flap, perforator failure, fascia lata, microsurgery

## Abstract

**Background:**

The anterolateral thigh (ALT) free flap is a workhorse in head and neck reconstruction, offering versatility, long pedicle length, and low donor site morbidity. Nevertheless, anatomical variability of its perforators can occasionally result in absent or unusable vessels, jeopardizing intraoperative feasibility. In such cases, harvesting an additional donor site increases operative time, morbidity, and aesthetic results. Salvage solutions within the same vascular territory are therefore essential to ensure safe and efficient reconstruction.

**Case presentation:**

We report a 70-year-old female with oral squamous cell carcinoma of the right retromolar triangle requiring composite resection with flap reconstruction. A patient-specific reconstructive plate and ALT free flap were planned. Intraoperatively, no usable perforators of the descending branch of the lateral circumflex femoral artery were identified. Proximal dissection revealed reliable perforators supplying the tensor fascia lata (TFL) territory, allowing elevation of a TFL free flap through the same incision. Microvascular anastomosis was performed successfully. The postoperative course was uneventful, and the flap remained viable throughout follow-up.

**Conclusion:**

This case highlights the importance of intraoperative flexibility when encountering ALT perforator absence. The TFL free flap represents a dependable salvage solution, characterized by more consistent vascular anatomy and minimal additional donor site morbidity. By enabling conversion within the same surgical field, the TFL flap reduces operative time and avoids the drawbacks of a second donor site. Incorporating this strategy into a structured decision-making algorithm enhances reconstructive safety and broadens the armamentarium for complex head and neck reconstruction.

## Introduction

The anterolateral thigh (ALT) free flap as a reliable soft tissue graft, is suitable for extensive head and neck reconstructions, is easy to harvest, and allows for a two-team approach to accelerate surgery (1). The flap typically has a predictable cutaneous perforator that allows the graft to be elevated in a variety of ways: as a fasciocutaneous flap, a musculo-fasciocutaneous flap, or a muscular flap (2). The anterolateral thigh (ALT) flap is widely used in reconstructive microsurgery, but anatomical variability of its perforators, reported in up to 23.6% of cases, may occasionally result in absent or unusable vessels, leading to intraoperative difficulties (3).

Despite the generally consistent anatomy of the lateral circumflex femoral artery (LCFA), intraoperative absence of a reliable perforator may occasionally be encountered, as preoperative angiography has limited predictive value in this setting (4, 5). When this occurs, two main strategies exist: either to identify an alternative local or regional flap within the same vascular territory, or to proceed with a second donor site, which inevitably increases morbidity and introduces additional aesthetic and surgical limitations (3, 6). In this case, arising local solutions include the anteromedial thigh flap, a proximally based ALT flap, the perforator-free ALT flap, the vastus lateralis muscle flap, the rectus femoris flap, and the tensor fascia lata (TFL) flap (7–10). Subsequently, intraoperative absence of a suitable ALT perforator can abruptly jeopardize the reconstructive plan, and therefore surgeons must therefore be prepared to adapt immediately to avoid reconstruction failure (4, 5).

When such a scenario occurs, the consequences for both the surgical workflow and the patient can be significant. Preparing and harvesting a second donor site prolongs the duration of surgery and requires typically modifications to the operative setup. Beyond these immediate intraoperative challenges, the use of an additional donor site carries the risk of higher morbidity, prolonged recovery and a greater likelihood of wound complications (1).

In this study, we present a clinical case in which no skin perforators could be identified during ALT flap elevation, and the intraoral defect was successfully reconstructed by conversion to a tensor fascia lata (TFL) free flap. In addition, we propose a practical decision-making algorithm to guide intraoperative management when ALT perforator failure is encountered.

## Case presentation

The patient, a 70-year-old female, first presented to our department with a biopsy-proven G2 oral squamous cell carcinoma (OSCC) of the right retromolar triangle with infiltration of the mandibular bone. Following initial presentation with histopathological confirmation, the case was discussed in the certified interdisciplinary tumor board of the General Hospital of Dortmund, which recommended composite resection and immediate reconstruction. As part of staging, contrast-enhanced CT scans of the head and neck, thorax, and upper abdomen were performed, which revealed no lymph node involvement or distant metastases. Preoperatively, the ALT donor site was examined with duplex ultrasound to identify suitable perforators. Surgery was scheduled and performed, consisting of tumor resection with mandibular reconstruction using a patient-specific plate (PSI; Stryker CMF) and a free flap reconstruction. An ALT free flap was initially planned as the reconstructive option.

**Figure 1:**
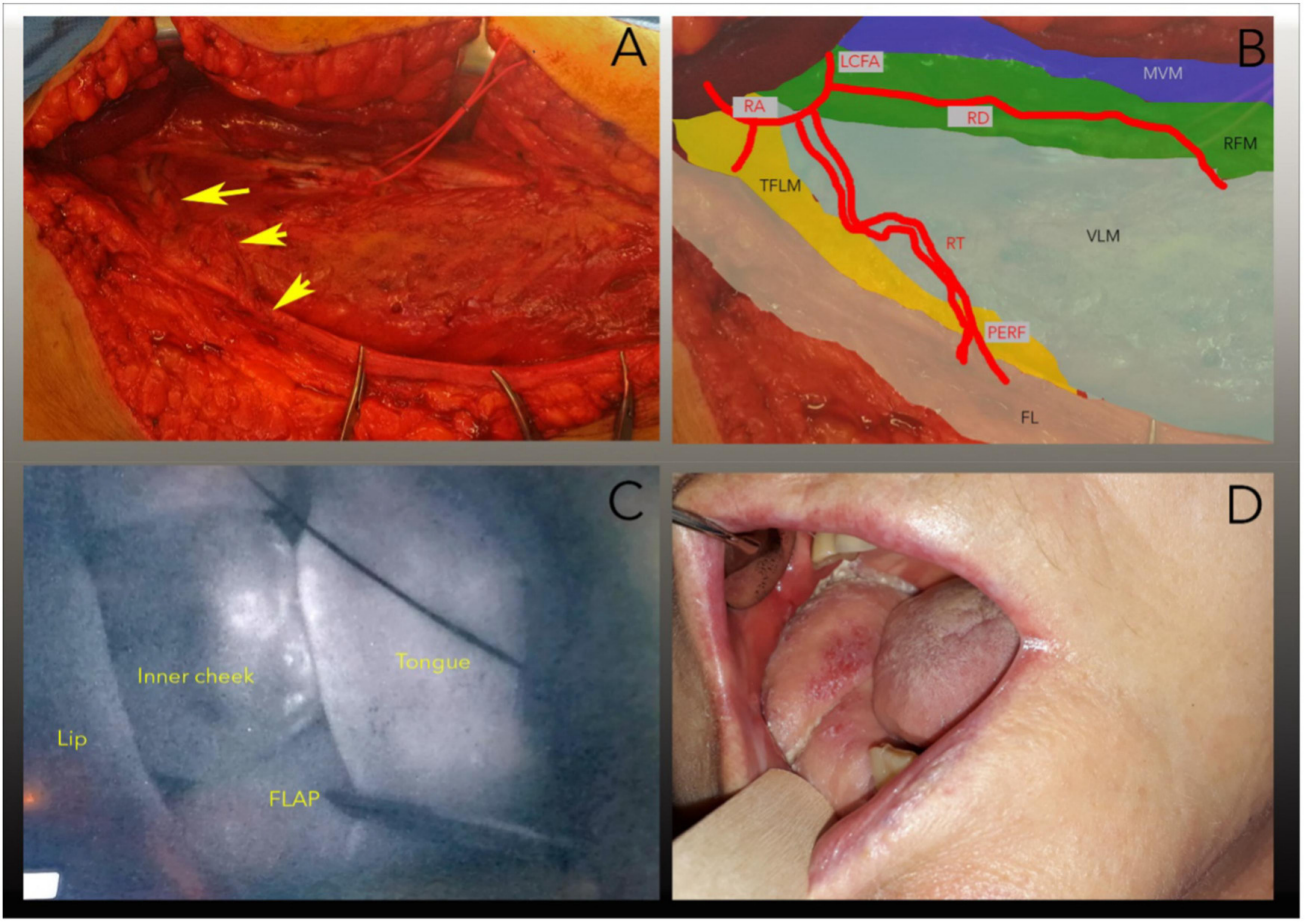
Illustration of the intraoperative situs. The arrows mark the double perforating vascular bundle (ramus transversalis) to the fascia lata. The vascular bundle holds the descending ramus of the lateral circumflex femoral artery. **(B)** Schematic representation of the intraoperative situation: RD, ramus descendens; RT, ramus transversus; RA, ramus ascendens; LCFA, lateral circumflex femoral artery; PERF, perforating branches of the transverse branch in the fascia lata; MVM, musculus vastus medialis; LVM, musculus vastus lateralis; TFLM, musculus tensor fasciae latae; FL, fascia lata, **(C)** Screenshot of the intraoperative indocyanine green (ICG) perfusion test. Note the evenly distributed fluorescence. **(D)** 4-week follow-up.

R0 resection was performed under frozen section control leaving a defect of 8 × 6 cm. The final histopathologic diagnosis was pT4a N0 Pn0 L0 V0 G2 OSCC according to UICC 2020. The operation was performed in a two-team approach, one team doing the tumor resection and one harvesting the ALT flap. Intraoperatively, no skin perforators of the descending branch of the LCFA were identified during careful dissection at 2.8× magnification. Proximal dissection revealed that all branches of the LCFA ascended from a proximal anastomotic ring (as shown in Figure 1B), including two strong perforators of the lateral branch that perforated the tensor fascia lata muscle, allowing the ascent of a fasciocutaneous flap for the planned reconstruction. The two lateral branches could be dissected along with a portion of the anastomotic ring, allowing for safe tissue transfer. Microvascular reanastomosis was performed with 9-0 Ethylon suture (Johnson & Johnson) using a Pentero surgical microscope (Zeiss). After successful tissue transfer, blood circulation was monitored with intravenous indocianine green solution (0.3 mg/kg) using the integrated fluorescence camera of the surgical microscope. Postoperative follow-up was uneventful. The flap remained viable with satisfactory healing at the recipient site, and no donor-site morbidity was observed. At four weeks, intraoral healing was complete. Clinical follow-up continued at three and six months, confirming stable flap integration, adequate mandibular contour with plate reconstruction, and absence of complications. The patient reported acceptable oral function for mastication and speech, with gradual improvement over time, and expressed overall satisfaction with the reconstructive outcome. No adverse or unanticipated events were recorded. Figure 1A shows the intraoperative situs, 1B the schematic drawing of the vascular anatomy, 1C the blood circulation under indocianine green fluorescence imaging, and 1D the clinical situation after a four-week healing period.

## Decision making algorithm

**Figure 2:**
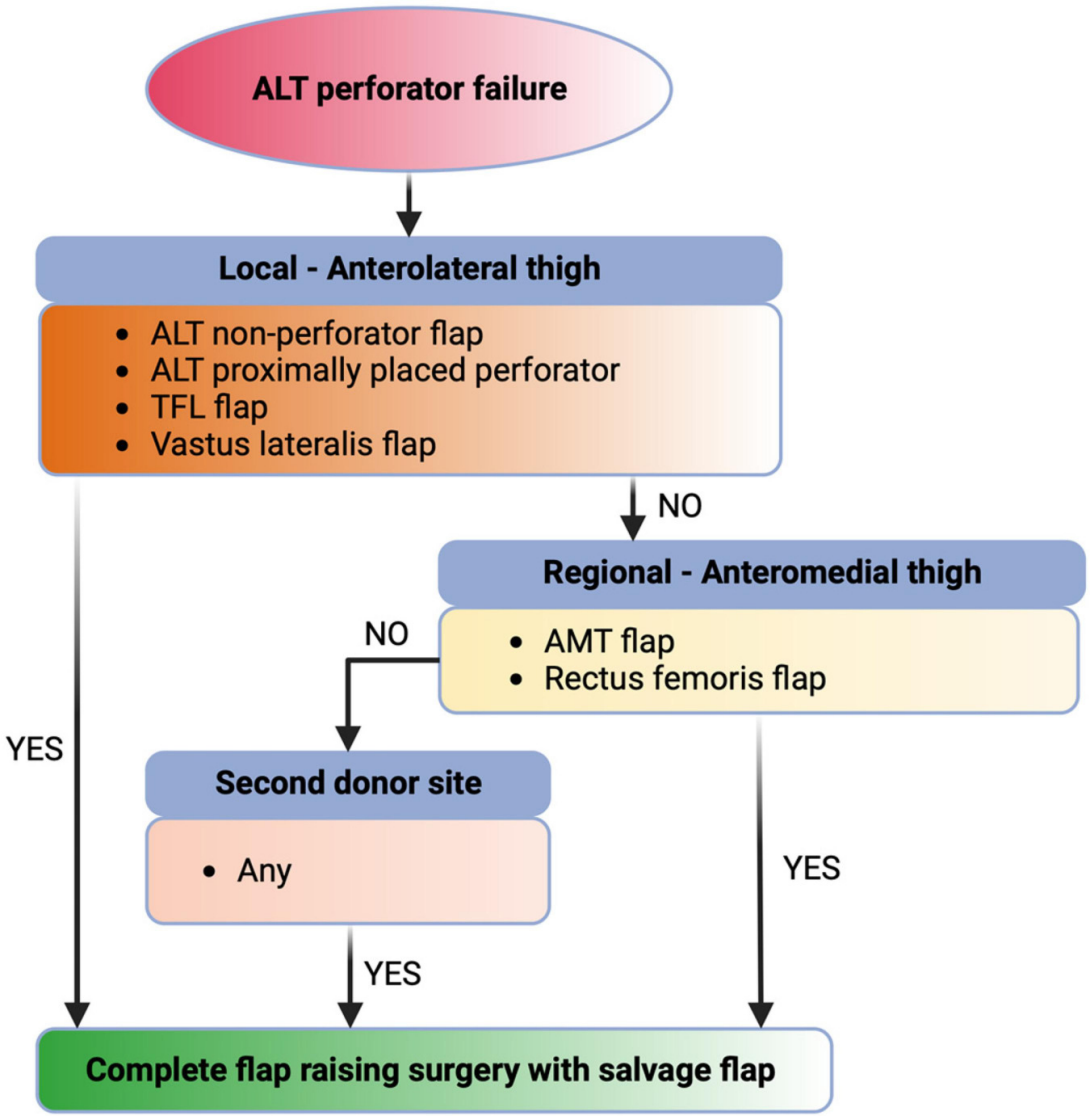
Illustration of the intraoperative decision algorithm for ALT skin perforator failure with possible options for flap salvage. TFL, tensor fascia lata; AMT, anteromedial thigh.

To facilitate the intraoperative decision in case of ALT skin perforator failure, we have introduced the following decision algorithm (Figure 2) based on the suggestions of Thomas et al, Hsieh et al. and our institutional experience (3, 11):

1. Local lateral approach: Dissection of the vascular pedicle is continued proximally to expose the vascular anatomy of the descending, oblique, transverse, and ascending branches of the deep circumflex femoral artery. If a more proximal perforator is found, it is used as the vascular pedicle and, if possible, a TFL flap is elevated.
2. Local medial approach: If adequate pedicles are not available, the anteromedial femoral flap is exposed and elevated if possible.
3. Second donor site approach: If a local flap cannot be elevated, the donor site is changed.

## Discussion

The anterolateral thigh (ALT) free flap is a well-established option for reconstruction, combining versatility, long pedicle length, and low donor site morbidity (7, 10). A consistent concern, however, is the anatomical variability of its perforators in terms of number, caliber, and course (2, 6). This variability may lead to intraoperative difficulties, and in some cases to non-viable flaps when no sizable perforators are identified (7, 12). In addition, the frequent presence of long musculocutaneous perforators increases the risk of iatrogenic injury during dissection, which can further compromise flap reliability (4, 5).

The absence or inadequacy of ALT perforators has been reported in 2.6% of planned reconstructions in a large multicenter head and neck series while iatrogenic injury account for 0.6% of the harvesting failures in the same study (3). Systematic reviews have documented a prevalence ranging from ∼0.89% up to 11.2%, highlighting significant heterogeneity between studies and patient populations while an anatomic meta-analysis estimated 1.8% of cases without any perforators (2, 8). Preoperative Computed Tomography Angiography (CTA) may assist in donor thigh selection but does not eliminate the risk of long, tortuous intramuscular perforators that remain vulnerable to iatrogenic injury during dissection or perforators that are of small caliber and therefore unreliable (4). More specifically, conventional CTA has shown limited predictive accuracy for mapping ALT perforators, with reported sensitivity of 27.7% and overall accuracy of only 36.7%, whereas catheter-based CTA performed better but is impractical for routine use (5). Even with modern imaging, the inherent anatomic variability means that intraoperative absence of usable perforators remains an unpredictable risk (6).

Within this context, the availability of reliable intraoperative salvage options is of critical importance. When the ALT flap proves non-viable due to absent or unsuitable perforators, choosing an alternative flap from the same donor thigh avoids the need for a second donor site and thereby reduces morbidity (7, 10). Local anterolateral thigh–based solutions include harvesting an ALT flap on more proximal perforators if available (10), conversion to an ALT flap based on small non-sizable perforators (12), or utilizing adjacent flaps supplied by the same vascular axis such as the tensor fascia lata (TFL) or vastus lateralis (8, 13). Regional anteromedial thigh–based alternatives include the anteromedial thigh (AMT) flap and the rectus femoris flap, both of which can be harvested through nearby access and also rely on the lateral circumflex femoral artery system (8, 9). Among these options, the TFL flap stands out for its consistent perforator anatomy and ability to be dissected through the same incision, offering a practical and reliable salvage solution with limited additional donor site morbidity (1, 7).

The tensor fascia lata (TFL) flap represents a dependable salvage solution when the ALT flap is not feasible. Its vascular anatomy is more constant, with multiple perforators reliably arising from the transverse branch of the lateral circumflex femoral artery, in contrast to the variability seen in ALT (11). Several series have confirmed that the TFL can provide skin paddles of adequate size for head and neck reconstruction, and its tissue can be safely thinned to achieve satisfactory contour (1). Historically, Song et al. first described the ALT flap in 1984 and highlighted its anatomical relation to the lateral thigh territory, which later facilitated the recognition of the TFL region as a potential donor site (14). More recent anatomical and clinical studies have emphasized that the TFL consistently contains robust perforators, with pedicle lengths of 7–8 cm and skin paddles extending up to 28 × 17 cm, comparable to or larger than conventional ALT designs (7). Jaiswal et al., reporting a total of 29 patients further demonstrated that the TFL is not limited to salvage situations but can also be combined with ALT in conjoint or chimeric configurations to reconstruct large, multidimensional oromandibular defects, broadening its reconstructive utility (1). In our case, the resection resulted in a defect of 8 × 6 cm, for which an ALT flap had initially been designed. Due to the absence of suitable perforators, the plan was converted intraoperatively to a TFL flap harvested through the same incision. The TFL provided a comparable skin paddle, sufficient to achieve tension-free closure and satisfactory contour.

Beyond the TFL, several other flaps from the same vascular territory have been described as salvage solutions when ALT perforators are absent or unusable. These include the anteromedial thigh (AMT) flap, proximally based ALT flaps, perforator-free ALT designs, as well as muscle-based options such as the vastus lateralis and rectus femoris (3, 7, 12). Each of these alternatives carries distinct anatomical and functional considerations, and their use is determined by intraoperative findings, defect requirements, and surgeon preference. However, no comparative study directly evaluating these options against one another is currently available.

In line with these reports, our case demonstrates successful intraoperative salvage of an abandoned ALT harvest with a TFL free flap, resulting in stable reconstruction without additional morbidity. This highlights the practical role of the TFL as a reliable fallback option within the reconstructive algorithm. Nevertheless, the conclusions from a single case must be interpreted with caution and require confirmation from larger series.

In summary, while the ALT free flap remains a cornerstone in reconstructive microsurgery, its inherent perforator variability continues to pose intraoperative challenges. The ability to transition seamlessly to salvage options from the same vascular territory is essential to avoid failed reconstructions. Among these, the TFL flap emerges as a particularly reliable solution, supported by strong clinical evidence. Incorporating such strategies into the reconstructive algorithm not only safeguards against intraoperative uncertainty but also broadens the armamentarium of safe and effective options for complex head and neck reconstruction.

## Data Availability

The original contributions presented in the study are included in the article/Supplementary Material, further inquiries can be directed to the corresponding author.
